# Hypnotherapy for procedural pain, itch, and state anxiety in children with acute burns: a feasibility and acceptability study protocol

**DOI:** 10.1186/s40814-022-01017-z

**Published:** 2022-03-09

**Authors:** Dali Geagea, Bronwyn Griffin, Roy Kimble, Vince Polito, Devin B. Terhune, Zephanie Tyack

**Affiliations:** 1 Centre for Children’s Burns and Trauma Research, Centre for Children’s Health Research, Level 7, 62 Graham Street, South Brisbane, QLD 4101 Australia; 2 Faculty of Medicine, The University of Queensland, Brisbane, QLD 4067 Australia; 3 Faculty of Health, School of Nursing, Queensland University of Technology, Kelvin Grove, QLD 4058 Australia; 4 Pegg Leditschke Paediatric Burns Centre, The Queensland Children’s Hospital, South Brisbane, QLD 4101 Australia; 5 School of Psychological Sciences, Macquarie University, Macquarie Park, NSW 2109 Australia; 6 Department of Psychology, Goldsmiths University of London, London, SE14 6NW UK; 7 Australian Centre for Health Services Innovation (AusHSI) and Centre for Healthcare Transformation, Queensland University of Technology, Kelvin Grove, QLD 4059 Australia

**Keywords:** Children, Hypnotherapy, Procedural pain, Anxiety, Itch, Acceptability, Implementation

## Abstract

**Background:**

Burns and related procedures are painful and distressing for children, exposing them to acute and chronic sequelae that can negatively affect their physiological, psychological, and social functions. Non-pharmacological interventions such as distraction techniques are beneficial adjuncts to pharmacological agents for procedural pain, state anxiety, and itch in children with burns but have limitations (e.g. lack of research on burn-related itch, tailoring, and consensus on optimal treatment). Hypnotherapy is a non-pharmacological intervention that can be tailored for varied settings and populations with evidence of benefit for itch and superior effectiveness in comparison to other non-pharmacological interventions for children’s procedural pain and state anxiety. Thus, children with burns can benefit from hypnotherapy as an adjunct to pharmacological agents. Yet, in paediatric burns, rigorous studies of effectiveness are limited and no studies have been identified that screen for hypnotic suggestibility, an important predictor of hypnotherapy outcomes. Considering potential barriers to the delivery of hypnotherapy in paediatric burns, the proposed study will examine the feasibility and acceptability of hypnotic suggestibility screening followed by hypnotherapy for procedural pain, state anxiety, and itch in children with acute burns.

**Methods:**

An observational mixed-methods feasibility and acceptability study will be conducted over 15 weeks. Eligible children (*N* = 30) aged 4 to 16 years presenting to a paediatric burns outpatient centre in a metropolitan children’s hospital in Australia with acute burns requiring dressing changes will be included. Eligible parents of children (*N* = up to 30) and clinicians who perform dressing changes (*N* = up to 20) will also be included. Child participants screened as having medium to high suggestibility as assessed by behavioural measures will receive hypnotherapy during dressing changes. A process evaluation will target feasibility and acceptability as primary outcomes and implementation (i.e. fidelity in delivery), reach, potential effectiveness, and adoption of evaluation procedures and intervention as secondary outcomes.

**Discussion:**

Ethical approval was obtained from the Queensland Children’s Hospital and Health Service ethics committee. Results will be published in peer-reviewed publications and conference proceedings.

The findings will guide the design of future trials on the effectiveness of hypnotherapy and inform the development of child-centred hypnotic interventions in children with burns.

**Trial registration:**

Australian New Zealand Clinical Trials Registry ACTRN12620000988954

**Supplementary Information:**

The online version contains supplementary material available at 10.1186/s40814-022-01017-z.

## Background

Acute paediatric burns pose a major problem to the health of children affecting yearly more than half a million persons below the age of 20, one-quarter of whom are below the age of 16 years [1]. Burns and concomitant treatments can cause pain, anxiety, and itch for children [2]. In the acute phase of treatment, pain, anxiety, and itch can aggravate each other, increase the inflammatory response, reduce adherence to treatment, and prolong the recovery process [2]. In turn, subsequent relapse and/or delayed recovery can lengthen hospitalisation time, augment medication requirements, and inflate healthcare costs [2]. Furthermore, inadequately treated pain, anxiety, and itch elicited by burns and related procedures can cause chronic sensory alterations (e.g. hyperalgesia, persistent pain) and psychosocial sequelae (e.g. psychopathologies; social, schooling, and sleep problems) [2]. These sequelae can increase the need for medications during subsequent procedures and may have a devastating effect on children’s well-being and quality of life. Treating post-burn itch, procedural pain, and state anxiety can prevent related biopsychosocial sequelae (e.g. post-traumatic stress disorder) and the impact of these sequelae on families as well as enhance re-epithelisation [2].

Procedural pain, state anxiety, and itch are usually treated in children with burns using non-pharmacological and pharmacological interventions [3]. According to a review of systematic reviews, hypnotherapy and distraction are supported by the most robust evidence of efficacy for paediatric procedural pain and distress in comparison to other non-pharmacological interventions [4]. Distraction techniques are among the most investigated and popular non-pharmacological interventions in paediatric burns [3]. Yet, evidence regarding distraction techniques is lacking for burn-related itch [5]. Plus, the high cost of virtual reality or multimodal devices, the need for training and technical support, and the lack of tailoring and consensus on the optimal method of delivery and technology may limit the use of distraction techniques [6, 7].

Hypnotherapy has a long history of use in the treatment of children’s physical and psychological problems [8]. A recent systematic review and meta-analysis showed that hypnotherapy can be effective in targeting both the affective and sensory components (i.e. situational determinants) of pain [9]. Neurophysiological studies have indicated that the effects of hypnotherapy on pain intensity and unpleasantness are via modulating the activity in the anterior cingulate cortex and increasing connectivity between cortical and subcortical areas [10]. Hypnotherapy may also be beneficial for treating burn-related itch as well as itch-related cognitive and emotional consequences, including distress, discomfort from itching skin, and scratching habits [11]. Although research has been predominantly conducted in adults, hypnotherapy may be more beneficial for children due to characteristics that make them more receptive to suggestions (higher suggestibility, fantasy proneness, engagement in play, and motivation to learn new skills) [8]. Systematic reviews indicate that hypnotherapy can be effective and potentially superior to standard care, control conditions, and other non-pharmacological interventions (e.g. distraction) in decreasing children’s procedural pain and state anxiety across a range of conditions [7, 12, 13]. A meta-analysis on hypnotherapy for procedural distress found larger effect sizes in children than adults adding to the potential importance of investigating hypnotherapy in children [12].

Hypnotherapy can be easily adapted to diverse settings and tailored to children’s different cognitive levels, preferences, and characteristics [14]. Due to adaptability and minimal technical requirements, hypnotherapy can be delivered within a short period (e.g. 15 min), without a hypnotherapist (self-hypnosis), and in varied delivery modes (live or pre-recorded) [14]. Yet, the delivery and outcomes of hypnotherapy may be influenced by potential barriers related to the paediatric burns setting (distressing nature, short time available to prepare children for medical procedures, possible interruptions), clinicians, and parents [14, 15]. For instance, mixed opinions among clinicians and misconceptions regarding hypnotherapy have been identified in the general population including Australia [14, 16]. Due to the unique biopsychosocial impact of burns, pain elicited by burns and related procedures may adversely affect children’s attitude and compliance and thus the applicability of delivering hypnotherapy and treatment outcomes [2, 15, 17]. Thus, investigating the feasibility and acceptability of hypnotherapy is paramount to guide research and clinical practice in children with burns [14].

Although research has indicated the benefits of hypnotherapy for children with a range of conditions, data is limited in paediatric burns. Only one recently conducted randomised controlled trial (RCT) on hypnotherapy for pain and anxiety in children with burns has been identified in a recent systematic review [6]. The RCT involving some of the current authors indicated that parents of children with acute burns expressed more satisfaction with hypnotherapy in comparison to standard care at the 3rd dressing change [18]. This finding parallels a meta-analysis in adults undergoing medical procedures or surgery in which shorter hospital stays, fewer medication requirements, and greater patient satisfaction leading to more cost-savings were linked to a hypnotherapy intervention delivered by clinicians [19]. Despite evidence supporting hypnotic suggestibility (i.e. degree of responding to hypnotic suggestions) as a predictor of hypnotherapy’s pain and anxiety outcomes in children, feasibility data on hypnotic suggestibility screening are lacking in children with burns [20]. Before investigating the efficacy of hypnotherapy in children with burns further, it is important to investigate the feasibility and acceptability of the intervention in an acute study setting.

## Methods

Reporting and methodology for the proposed study follow the Standard Protocol Items Recommendations for Interventional Trials (SPIRIT 2013) [21], Standards for Reporting Implementation Studies initiative (StaRI) [22], as well as Consolidated Standards of Reporting Trials (CONSORT) - extension to randomised pilot and feasibility trials [23], and Strengthening the Reporting of Observational Studies in Epidemiology (STROBE) [24] (supplementary file 1). The intervention has been reported using the Template for Intervention Description and Replication (TIDieR) [25].

### Study aims and objectives

The primary aims are to investigate the feasibility and acceptability of the intervention (i.e. pre-hypnosis interview, hypnotic suggestibility screening, and hypnotherapeutic session) and evaluation procedures (i.e. recruitment and health outcome data collection). Additionally, the reach, adoption, potential effectiveness, and implementation (i.e. fidelity) of the intervention and evaluation procedures will be examined as secondary outcomes.

### Patient and public involvement

Patients or the public were not directly involved in the study development. A previous study of hypnotherapy involving children with burns and their parents was used to inform the study design [18].

### Design

The study will be conducted using an observational mixed-methods design integrating multiple data sources (clinicians, parents, and children) and data collection methods (quantitative and qualitative) [26]. The use of different perspectives and methods is intended to avoid biases generated by relying on one source of evidence or type of data (e.g. quantitative or qualitative alone). The key outcomes are acceptability (i.e. the extent to which procedures are perceived as fit, satisfying, and appealing, based on experienced affective and cognitive reactions) [27], implementation (i.e. fidelity or the extent to which procedures are delivered as intended), and feasibility (i.e. the extent to which procedures are successfully delivered in a distinctive context that is not fully controlled) [28]. The Theoretical Framework of Acceptability (TFA) [27], Outcomes for Implementation Research [28], and the Reach, Effectiveness, Adoption, Implementation and Maintenance (RE-AIM) framework were used to guide the process evaluation [29–31] (Table 1).

**Table 1:** Evaluation frameworks guiding the process evaluation and data collection

Evaluation framework	Implementation outcome	Definition		Data collection	
				Source	Type
RE-AIM [30, 31]	Reach^a^	Willingness of eligible families to participate in the study		Report on the rate of approached eligible families who consent to participate	Quantitative
	Effectiveness	Extent to which the intervention eliciting intended effects and the overall perceived benefits and potential undesired effects		Numeric ratings of pain, anxiety, and itch; physiologic measures of distress; data on wound re-epithelialisation	Quantitative
				Reported adverse events	Qualitative
				Reported perceived benefits	Qualitative
	Adoption by clinicians^b^	Willingness of clinicians to take part in the study		Report on the rate of clinicians conducting dressing changes who consent to join the study	Quantitative
	Implementation	The quality of delivery and consistent delivery of study procedures as intended or prescribed in the protocol (i.e. fidelity)		Report on retention rates among children	Quantitative
				Level of adherence to the fidelity checklist in delivering the intervention	Quantitative
				Completeness of collected data on health outcomes	Quantitative
				Additional adaptations and modifications	Qualitative
	Maintenance	Degree of long-term outcomes by participants or program sustainability within the setting		Not assessed due to the absence of follow-up	
TFA [27]	Acceptability	Perceived self-efficacy	Confidence in the ability to accomplish the behaviour(s) needed to participate in the intervention	Level of children’s perceived self-efficacy on a Likert scale	Quantitative
		Ethicality	Level of understanding of the intervention	Level of children’s positive therapy expectations on a Likert scale	Quantitative
		Coherence	Degree to which the intervention fits well with participants’ beliefs and value system	Semi-structured interview on beliefs and views of children and parents on hypnotherapy	Qualitative
		Perceived effectiveness	The degree to which the intervention is perceived as likely to succeed in its purpose	Families’ and clinicians’ satisfaction with hypnotherapeutic sessions and study procedures (procedures are not rated by clinicians) on NRS	Quantitative
				Semi-structured interview with parents, children, and clinicians on likes, dislikes, and recommendations	Qualitative
		Perceived burden	Perceived extent of effort required to engage in the intervention	Semi-structured interview on perceived required time and cognitive effort for the intervention	Qualitative
OIR [28]	Feasibility	The extent to which study procedures can be successfully delivered to participants in a distinctive context that is not fully controlled		- Number of disruptions - Available time (recordings)	Quantitative
				Field notes on the adequacy of resources	Qualitative

*TFA* Theoretical framework of acceptability, *NRS* Numeric Rating Scale, *OIR* Outcomes for implementation research

^a^*Representativeness* (i.e. the similarity between participants and eligible patients) will not be assessed as part of *Reach* due to the small sample size [31]

^b^Adoption at setting level is already established

### Setting

The study will be conducted in a specialist paediatric burns centre in a metropolitan children’s hospital in Australia.

### Participants

Children presenting to the study setting with acute burns requiring dressing changes, who agree with their parents to be contacted for research, will be recruited after reviewing their medical files and assessing their eligibility as advised by clinicians. Eligible children will be included with their parents and clinicians if they agree to participate. Children’s participation involves providing data and engaging in the intervention. Parents’ participation involves providing proxy-completed outcome data for children under 8 years and acceptability data. Clinicians’ participation involves rating their satisfaction with hypnotherapeutic sessions, reporting adverse events, and advising researchers on eligible families who agree to be contacted for research.

#### Sample size

A formal sample size calculation is not required for feasibility studies [32]. Hypnotherapy will be conducted with 30 children over 15 weeks. The sample size is estimated based on clinic data in the study setting indicating that at least five potentially eligible children present with acute burns each week. Considering a non-participation rate of 15% of the potentially eligible children and the exclusion of 15% of children with low hypnotic suggestibility, it is estimated that 15 weeks will be required to recruit at least 30 eligible children. This number of participants is estimated to be adequate to generate enough information on the feasibility and acceptability of the intervention and study procedures. Participants will be followed up for approximately 5 weeks as the average number of dressing changes is five (one dressing change per week). The participation rate and recruitment time will inform sample size calculations in future studies.

#### Eligibility criteria

Children will be eligible if they have an acute burn injury of any depth (except superficial burns), are aged from 4 to 16 years, and speak English. Children will be excluded if they have deafness, cognitive impairment, a diagnosed severe psychiatric disorder, involvement with child safety or disability services, need for ventilator support, general anaesthesia requirement for their first dressing change, or a large total body surface area (TBSA >10%). Parents of children will be eligible if they speak English. Clinicians will be eligible if they are responsible for changing child participants’ burns dressings during the study period.

##### Special considerations

Despite the absence of agreement on contraindications to hypnotherapy, screening for patients with cognitive deficits or severe psychiatric disorders is important as they may not be responsive or comfortable with the experience [33]. These patients will be identified by clinicians based on comorbidities or routine psychosocial screening (i.e. collection of data on diagnosed psychiatric disorders) before being approached for recruitment.

### Intervention

Hypnosis is established with an induction procedure to enhance responsiveness and reduce peripheral awareness followed by delivering suggestions within a specific sociocultural context to guide participants to experience cognitive, sensory, motor, or perceptive alterations [34, 35]. Whereas the term *hypnosis* is used to describe the hypnotic process, *hypnotherapy* or *clinical hypnosis* refers to the therapeutic use of hypnosis in medical and psychotherapeutic contexts [8, 34, 35]. Hypnotherapy is thus defined as a treatment modality using suggestions in a hypnotic context to elicit perceptual, sensory, motor, and cognitive changes for therapeutic purposes [36]. The study intervention involves a pre-hypnosis interview followed by hypnotic suggestibility screening (during the first dressing change) and a hypnotherapeutic session.

#### Pre-hypnosis interview and hypnotic suggestibility screening

The pre-hypnosis interview and hypnotic suggestibility screening are intended to establish rapport, promote hypnotic responding, screen out irresponsive participants, and guide hypnotherapeutic sessions.

##### Pre-hypnosis interview

The interview is intended to clarify misconceptions about hypnotherapy; mitigate anxiety; enhance compliance, therapy expectations, and attitude; build rapport and trust; and collect information from children and parents to guide tailoring the hypnotherapeutic session [8]. The interview will be conducted based on literature regarding what can promote optimal therapy for children in pain (supplementary file 2 - table A) [8].

##### Hypnotic suggestibility screening

Hypnotic suggestibility, an important factor in response to hypnotic suggestions, will be screened for using the Stanford Hypnotic Scale for Children (SHCS-Children) immediately before children’s first dressing change (Table 2) [37]. Individuals with low hypnotic suggestibility are unlikely to benefit from hypnotherapy whereas those with high suggestibility show the strongest response to hypnotic analgesia and those with medium suggestibility have been shown to obtain pain relief [9, 38]. Therefore, only children with medium to high hypnotic suggestibility scores will be eligible for hypnotherapy to limit participant-related confounding factors by having more homogenous participants in terms of response, potentially increasing the effects of hypnotherapy. Children with low hypnotic suggestibility scores will be excluded from the study but will continue to receive standard care to avoid exposing children who are unlikely to obtain pain relief with hypnotherapy to an unnecessary burden of hypnosis (Table 2). Low scores (below three on the standard SHCS-Children and below two on the modified SHCS-Children) were determined based on the hypnotic suggestibility literature (Table 2). Due to the great variability in response to specific suggestions, individuals with high hypnotic suggestibility may be highly responsive to some suggestions and unresponsive to others [33]. Therefore, a tailored version of the hypnotic suggestibility  scale incorporating anaesthesia suggestions will be used to assess specific responses to hypno-anaesthesia suggestions and thus inform the delivery of therapeutic suggestions [33]. Responses to test suggestions of the hypnotic suggestibility scale will be used to guide the hypnotherapeutic session by informing the hypnotherapist on children’s preferences (i.e. favourite music type and memory) and the type(s) of suggestions they are responsive to [33].

**Table 2:** Description and psychometric properties of measures of behavioural and involuntariness responses to hypnotic suggestions

Measurement	Goal	Description	Score ranges	Scoring	Duration	Time point	Psychometric properties
Standard SHCS: CHILD form [37]	To measure behavioural responses to hypnotic suggestions for children aged 6–16 years	- Relaxation/eye closure induction; - 7 test suggestions for time regression, visualisation, ideomotor responses, and post-hypnotic suggestions to re-enter hypnosis at hand clap^a^	0–7: - Low: 0–2; - Medium: 3–5; - High: 6–7	- 0 if absent or negative behavioural response, - 1 if positive behavioural response [37]	20 mins	Just before and during the 1st dressing change	- Moderate correlation with the Stanford Hypnotic Susceptibility, Form A scale: *r* = 0.67 [37] - Established concurrent validity [37]
Modified SHCS: CHILD form [37]	To measure behavioural responses to hypnotic suggestions for children aged 4–8 years	- Active induction in which eyes can be open; - 6 test suggestions for time regression, visualisation, and ideomotor responses	0–6: - Low: 0–1; - Medium: 2–4; - High: 5–6				
Involuntariness numeric scale [39]	To rate involuntariness and automaticity experienced during the responses to SHCS: CHILD suggestions	Age-appropriate question reworded to children’s age regarding experienced involuntariness during responses to SHCS: CHILD items (*did that feel like you made that happen or that it happened by itself?)*^b^	Mean of items scores	Score ranging from 0 (no response), 1 (voluntary response to suggestions) to 5 (involuntary-automatic enactment) for each SHCS: CHILD test item	10 mins	During the 1st dressing change	- Good internal consistency (*α*s: 0.76–0.86) [40] - Adequate construct validity: moderate correlation of involuntariness scores with scores on a standardised hypnotic suggestibility scale (*r* = .49, *p* < .001, 95% *CI* = [.31, .65], *n* = 58) [41]

^a^Anaesthesia suggestion will be incorporated in the ideosensory test suggestion to assess specific responses relevant to hypno-anaesthesia

^b^Following each test suggestion, children will be asked about the involuntariness experienced during responses to test suggestions

#### Hypnotherapeutic session

Hypnotherapeutic sessions consist of induction, deepening (i.e. intensification) techniques, and therapeutic suggestions prior to counter-suggestions (Table 3). A maximum of five sessions will be provided during dressing changes that are usually performed every 3 days or weekly. Sessions will thus have the same duration as the burns dressing change, which is usually between 20 and 35 minutes. During the first dressing change, the session will be reduced to therapeutic suggestions following the standard induction included in the hypnotic suggestibility scale. Pre-recorded music will accompany sessions (supplementary file 2- table B) [8, 42]. External distractions will be minimised as much as possible with the hypnotherapist being the only person talking to the child whenever possible. Following clinical policies and to reduce separation anxiety, parents will be encouraged to be present during sessions, unless they choose not to do so [15].

**Table 3:** Description of the phases and components of the hypnotherapeutic session

Phase	Core components	Description of components	Goal
Induction	Positioning	Ask children to be in a comfortable position for hypnosis [8].	To promote comfort and relaxation [8].
	Eye fixation and abbreviated progressive muscle relaxation	Ask children to focus their attention on the hypnotherapist’s hand while tensing their hand’s muscles for a few seconds before releasing tensions [8].	To elicit relaxation, shift of attention, absorption in hypnosis, and dissociation from the painful stimulus [43, 44].
	Eye closure	Ask children to optionally close their eyes [8].	To block external distractions and promote internal absorption [37].
	Lifting of the hand	Ask children to keep their hands in a vertical position.^a^	To promote numbness in the hand and response expectancies during glove anaesthesia.
	Deliberate faster breathwork and suggestions for relaxation	Ask children to maintain faster and deeper rhythmic breathing for a few minutes then to gradually relax body muscles following pleasant imagery suggestions (e.g. *blowing tensions away and inhaling relaxing lights*) [8].	To enhance suggestibility by inducing mental and physical relaxation; promote positive coping by distraction; establish the hypnotic context; and promote response expectancies [8, 43, 44].
	Favourite place imagery	Give suggestions for favourite place imagery with few details that allow children to project their own experiences and preferences (e.g. *imagine your favourite place*) [8].	
	Ideomotor signalling	Ask children to make a physical movement (e.g. lifting a finger) as a sign of positive response to suggestions [8].	To reflect the inner experience of children, test response to suggestions and promote children’s sense of control [8].
	Metacognitive suggestions	Notify children on the nature of subsequent procedures or what is next in the intervention [63].	To promote children’s cooperation, positive response expectancies and prepare them for subsequent intervention steps [44].
Deepening/intensification suggestions	Deepening/intensification ideomotor suggestions	Give suggestions for movement without conscious muscle activity (e.g. eyelid catalepsy, arm heaviness) then test responses to suggestions [8].	To reinforce suggestions; accentuate dissociation from the external setting, focus of attention and absorption in the hypnotic experience [8].
	Compounding suggestions^b^	Give suggestions for absorption in hypnosis accompanying physical experience and sensations (e.g. *As your hand drops back down to your side, you can slowly and gently go deeper into hypnosis*) [8].	
	Counting and stairs imagery	Give suggestions for relaxation and absorption involving visualisation (e.g. *count out-loud as you go downstairs, go deeper into hypnosis*) [8].	
Therapeutic suggestions	Hypno-analgesia and hypno-anaesthesia suggestions	Deliver direct suggestions for ideosensory alterations (e.g. numbness and coolness) [8].	To reduce pain sensations by inducing analgesia and anaesthesia [8].
		Give indirect suggestions of pleasant imagery involving regression into enjoyable pain-free past events; glove-anaesthesia technique, metaphors for dissociation or substitution of pain-related sensations, cognitions, and feelings (e.g. darkness representing discomfort is transformed into a relaxing light) [8]. N.B. Suggestions for pleasant imagery can involve a detailed description of elements to visualise to assist imagination or fewer details that enable children to project their experiences and preferences (e.g. *imagine flowers that you like*) [8].	To target sensory, cognitive, and affective components of pain by respectively inducing sensory (analgesia, anaesthesia), perceptual (altered perception and expectations) and affective (agreeable feelings, reduced pain unpleasantness) alterations; accentuate dissociation from pain and absorption in pleasant internal experience; promote relaxation, comfort, positive change, and reduced alienation; guide the use of children’s internal resources for better coping and sense of control [8, 10].
	Suggestions for positive conditioning	Describe the setting as a place for healing rather than a source of distress and portray pain as a signal of injury to be treated rather than an enemy to be defeated [8].	To promote positive conditioning involving trust and positive views towards clinicians, treatment procedures, pain, and the medical setting [8].
Post-hypnotic suggestions	Anchoring	Use “anchoring techniques” and test the response to the anchor under-hypnosis (e.g., re-experiencing itch relief at hand clap) [8].	To teach children effective ways to reduce itch and distress while promoting autonomy, confidence, and positive coping [8].
	Self-hypnosis training	Give suggestions to practise self-hypnosis at home [8].	
	Future progression	Give suggestions for pleasant future imagery using children’s preferred element as a gateway to future events (e.g. *crossing a bridge to a pleasant future*) [8].	To promote positive conditioning with enhanced comfort, relaxation, and cooperation in future dressing changes; itch relief; enhanced healing [8].
Emergence from hypnosis	Counter suggestions	Give alerting suggestions while counting; then ask children to open their eyes and gradually switch to a seated position when ready after their awareness is back to the usual state (e.g. *at the count of five you can open your eyes, feeling alert*) [8].	To remove numbness and anaesthesia while inducing alertness and switching of awareness to the external surrounding [8].
Post-hypnosis phase	Post-hypnotic talk	Invite children to ask questions and discuss their experience; debrief those with negative reactions [8].	To promote safety, trust, and positive coping [8].
	Self-hypnosis tape and test of anchoring	Test children’s response to the “anchor” and give them a recording for self-hypnosis [8].	To reinforce therapy outcomes, positive coping, and sense of control [8].

^a^Children are asked to keep their hands in a vertical position throughout the session

^b^Compounding suggestions: continuous establishing of new responses onto the foundation of past responses

##### Addressing influencing factors

For optimal treatment, the intervention will address not only pain sensations but also biopsychosocial factors of hypnotic responding (e.g. views towards hypnotherapy, context, imaginative capacities, hypnotic suggestibility) (supplementary file 2 - table C). The intervention will also be tailored to situational (e.g. cognitive, emotional, attentional, and social components of pain) and predisposing (e.g. beliefs, cultural learning, cognitive level, and past conditioning) factors of pain (supplementary file 2 - table D) [2, 17, 45–48].

#### Delivery mode, material, and provider of the intervention

For optimal therapy, individual interventions will be provided face-to-face, not via audiotape to address changes in pain, distress, itch, and perceptions of hypnosis [8, 12]. Following the initial delivery of hypnotherapy, children will be provided with a taped recording of instructions for self-hypnosis for daily practice to reduce the time needed to teach them self-hypnosis. The provider of the intervention is a certified hypnotherapist and a member of the National Guild of Hypnotists. The hypnotherapist has extensive experience (more than ten years) in conducting hypnotherapy with children from diverse backgrounds with a range of conditions involving pain and distress in therapeutic and medical settings. Phases and components of the intervention are detailed in an intervention manual that can be obtained upon request.

#### Tailoring, adaptations, and fidelity in delivery

The hypnotherapist will adhere to the manual in delivering core elements of the intervention to facilitate replication and target procedural pain, state anxiety, and itch. The fidelity in delivering the intervention will be assessed using a fidelity checklist (supplementary file 2 - table E). The intervention will be tailored and adapted as informed by the pre-hypnosis interview and hypnotic suggestibility screening (supplementary file 2 - table F) to account for the hypnotherapist’s style, children’s preferences, and factors related to children, the setting, parents, and clinicians [8].

#### Concomitant treatments

Children will receive standard care including a combination of pain medications offered by treating clinicians at least 20 min (medications’ onset of action) before the dressing change in specific dosages that are part of usual care (supplementary file 2 - table G). Active (e.g. interactive toys including video games, virtual reality devices, controlled breathing, and guided imagery) and passive distraction techniques (e.g. passive music, videos on a portable device) will also be used as part of standard pre- or post-procedural care.

### Measurements

#### Hypnotic suggestibility and involuntariness

Child participants’ hypnotic suggestibility will be assessed by measuring behavioural responses to test suggestions using the Stanford Hypnotic Scale for Children [37]. The use of the scale is based on age-appropriate content for children between 4 and 16 years and administration in previous paediatric research (Table 2) [20, 37, 49]. Involuntariness will be measured to discern whether the responses to suggestions were due to simple compliance or higher responsiveness as the experience of involuntariness is central to the classic suggestion effect [39]. Bower’s numeric scale will be used to measure the degree to which children experience involuntariness during responses to test suggestions of the Stanford Hypnotic Scale for Children (Table 2) [39].

#### Sociodemographic data and burns characteristics

Data on child participants’ sociodemographic and burn characteristics will be gathered from clinicians, hospital charts, and medical records to describe the sample and triangulate data (Table 4).

**Table 4:** Collection of data on sociodemographic and clinical characteristics

Sociodemographic data and clinical characteristics	Dressing change time point			Source of data	Assessor	Dressing change
	Pre	Mid	Post			
Sociodemographic data: burn aetiology and site, ethnic background, comorbidities, skin type, and adjunct interventions (pain medications and first aid)			x	Patient interview	Treating surgeon or nurse	1st dressing change
Burn characteristics (burn depth, TBSA, mechanism, and site of injury)	x			Medical baseline examinations		

#### Process evaluation

Outcomes of the implementation process are outlined in Table 5. Potential effectiveness will be measured by health-related outcomes of procedural pain, state anxiety, and itch at different time points as guided by the RE-AIM outcome of *effectiveness* (Table 6). The selection of health outcomes measurement tools is based on psychometric properties, feasibility, and clinical utility for children aged from 4 to 16 years (supplementary file 3). Salivary α-amylase, a bio-indicator of the sympathetic adreno-medullary system (produced by norepinephrine-responsive salivary gland cells), will be measured as a stress biomarker. The choice of salivary α-amylase was made as the authors are experienced in administering the test rapidly in children research studies, and based on the measures’ reliability, validity, and sensitivity as a marker of distress and minor stressors [50].

**Table 5:** Process evaluation with data collection time-points

Implementation outcome	Measurement	Dressing change time point			Source of data (measurement tool)	Assessor	Dressing change
		Pre	Mid	Post			
Reach	Rate of families willing to participate out of those approached for participation	x			Report	Hypnotherapist	1st dressing change
Acceptability	Parents’ and children’s beliefs/views on hypnotherapy	x			Field notes, semi-structured pre-hypnosis interview^a^		
	Children’s level of perceived self-efficacy	x			1–10 Likert scale		
	Children’s level of positive therapy expectations	x			1–10 Likert scale		
	Satisfaction of parents and children with suggestibility screening, involuntariness measures, and pre-hypnosis interview			x	Satisfaction NRS^b^		
	Satisfaction of parents, children, and clinicians with the hypnotherapeutic session			x	Satisfaction NRS^b^		Each dressing change
	Families’ and clinicians’ likes, dislikes, recommendations, and perceived burden of required time and effort regarding hypnotherapeutic sessions			x	Semi-structured interview^a^ and field notes		Last dressing change
	Satisfaction of families with health outcomes measurements			x	Satisfaction NRS^b^		
Effectiveness	Perceived benefits			x	Field notes, reports of children, parents, and clinicians		
	Safety of hypnotherapy (rate, timing, duration, and severity of adverse events)		x	x			Each dressing change
	Health outcomes	(Refer to Table 6 for details)					
Adoption	Rate of clinicians consenting to participate among those conducting dressing changes.	x			Report	Hypnotherapist	1st dressing change
Implementation: fidelity	Retention rate		x	x	Report on the rate of children completing the intervention		Each dressing change
	Fidelity in collecting data on health outcomes			x	Response rate in data collection forms (amount and type of missing data)		
	Fidelity in delivering the intervention			x	Study-specific checklist^c^	Independent observer expert in clinical hypnosis	
	Additional adaptations and modifications			x	Documentation of what, when, and how adaptations occurred		
Feasibility	- Adequacy of resources (adequately working music player, calm space) - Presence/absence of interruption (s) - Time availability (sufficient for delivering study procedures) [31]	x	x	x	- Field notes - Intervention pre-recording	Hypnotherapist	

^a^Following permission from children and parents to audiotape the intervention

^b^Adult participants (parents or clinicians) and children ≥ 8years will self-report their satisfaction; proxy reports will be obtained from parents for children < 8years

^c^An independent assessor (preferably hypnotherapist) will assess the fidelity of delivering the intervention through analysing pre-recordings using a study-specific checklist based on the intervention manual; % of core elements and non-core elements delivered will be reported

**Table 6:** Health-related outcomes measured using the RE-AIM outcome of effectiveness with data collection time points

RE-AIM outcome	Health-related outcomes		Dressing change time point			Source of data (measurement tool)	Assessor	Dressing change
			Pre	Mid	Post			
Potential effectiveness	Acute procedural pain	Pain intensity	x	x retro	x	FPS-R (≤ 8 years) [51], I-INRS (≥ 8 years) [52], FLACC^a^ [53]	Hypnotherapist	Each dressing change
		Pain unpleasantness				UNRS (≥ 8 years) [52]		
	State anxiety		x	x retro	x	VAS [54]		
	Itch	Intensity			x	Itch-NRS: self-report ≥ 8 years, proxy-report < 8 years [55]		From 3rd dressing change
		Frequency				Questions on itch episodes (per week, per day) [55]		
	Physiologic measures of pain and distress	Heart rate	x	x	x	Monitoring device		Each dressing change
		Salivary α amylase (in children’s saliva samples)	x		x	ELISA kits (Stratech Scientific, Avalon NSW)	Independent observer	
	Wound healing	Duration and the total number of dressing changes until 95% re-epithelialisation	x	x	x	Medical records or reports of clinicians	Independent surgeon and nurse	
		% of re-epithelialisation		x				

*NRS* Numeric Rating Scale, *INRS* Numeric Rating Scale for Pain Intensity, *UNRS* Numeric Rating Scale for Pain Unpleasantness, *FPS-R* Faces Pain Scale-Revised, *FLACC* Face, Legs, Activity, Cry, Consolability, *retro* Retrospectively

^a^The FLACC scale will be used as a behavioural measure of child participants’ pain using nurse observation

### Data analysis and interpretation

Qualitative and quantitative data will be collected concurrently and subsequently analysed independently before integration.

#### Quantitative analysis

Descriptive statistics (frequencies, percentages, medians, and interquartile ranks) will be used to present data on children’s sociodemographic and clinical characteristics as well as process evaluation and health outcomes data. Suggestibility screening data will be stratified by age corresponding to the age groups of the two different forms of the Stanford Hypnotic Scale for Children that will be used in the study (4 to 6 years and 6 to 16 years). Data analysis will be conducted using Excel and SPSS 26 (IBM Corporation, Armonk, NY, USA).

#### Qualitative analysis and interpretation

Semi-structured interviews will be audio-recorded, transcribed verbatim, and analysed using framework analysis involving transcription, familiarisation, coding, developing a working thematic framework, charting of data into the framework, and interpretation of data [56]. Following familiarisation and coding of several interviews, qualitative data will be summarised by category and charted individually to a Microsoft Excel spreadsheet matrix to form a working framework [56]. After systematically indexing subsequent transcripts using the generated codes and categories of the framework, data will be interpreted within-and-between groups (children, parents, clinicians) to reach a consensus on themes. Themes will be generated using an inductive approach. Following the completion of the inductive coding, interview data will be mapped to the Theoretical Framework of Acceptability deductively [27]. The credibility of the analysis will be maximised using member checking, independent coding by two researchers, triangulation of the results across participant groups (e.g. parents and children), and reflexivity. Reflexivity involves keeping notes of what occurs during the interview (field notes), early data interpretations, as well as ideas and impressions by researchers conducting the interviews and analysing the data [56]. Within-group triangulation of interview data will be conducted using emergent themes to identify differences according to child, parent, or clinician report [26]. The strength of convergence will be assessed based on the frequency and range of overlapping themes. Themes will be rated across participant reporting groups to identify those with the highest versus the minimum convergence. Differences in evidence across the groups will be reported.

### Data synthesis

A transformative approach to data synthesis will be adopted to integrate different sources and types (qualitative and quantitative) of evidence through triangulation and interpretation based on the implementation outcomes [28]. For instance, acceptability will be assessed using interview data as well as ratings of families and clinicians’ satisfaction, children’s self-efficacy, and positive therapy expectations. Some qualitative data will be merged into quantitative counts. For instance, the number of times recurrent themes related to acceptability emerge in interviews will be counted. Following the triangulation of data, each implementation outcome will be allocated a score (1 = less successful, 2 = moderately successful, 3 = highly successful) based on the availability of data addressing the implementation outcome and the positivity of outcome measures (supplementary file 4).

### Procedures

#### Recruitment

Children presenting to the specialist burns centre for their first burns dressing change and their parents will be screened for eligibility after consulting with clinicians regarding children’s potential eligibility. It is anticipated that approximately three children per week will be recruited (total recruitment period 15 weeks).

#### Intervention phase

The hypnotherapist will conduct a semi-structured interview with families following their consent to participate. Saliva samples will be collected immediately before medications at least 20 minutes before removing the burns dressing by placing Salivettes® (Sarstedt Australia Pty Ltd, Mawson Lakes, S.A., Australia) under children’s tongues for 2 minutes. Data on burns characteristics and health outcomes (pre-procedural pain, state anxiety, and heart rate) will be collected at baseline. Hypnotic suggestibility screening will be conducted immediately before the first dressing change to measure behavioural responses to hypnotic suggestions. Children who score low on the hypnotic suggestibility test will be guided to emerge from hypnosis and excluded from the study, whereas those with medium to high scores will receive therapeutic suggestions until the new dressing is applied.

#### Post-intervention

Saliva samples will be collected by the hypnotherapist 10 minutes following dressing changes. Information will be recorded regarding children’s medications, time of last waking up, the last brushing of teeth, food/drink/gum intake during the previous hour, and caffeine consumption during the collection day. The date and time of collection and the volume of saliva samples will be recorded. Samples will be stored at 4°C to be spun in a centrifuge at 1400 × *g* for 10 min at room temperature and then frozen at −80°C until analysis in triplicate within 7 days. Following the collection of data on sociodemographic characteristics and process evaluation outcomes, data analysis, and synthesis will be performed.

### Study timeline

**Fig. 1:**
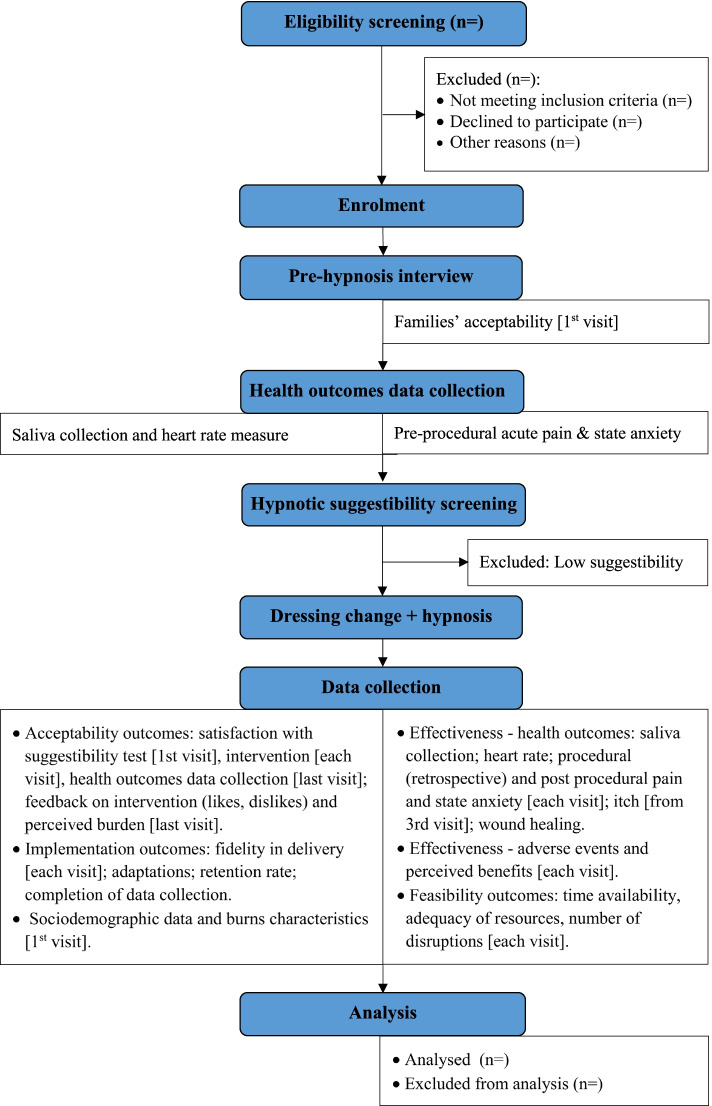
Flow diagram describing the timeline of study procedures

The study will commence in March 2022 and is expected to be conducted over 15 weeks as shown in the flow diagram (Fig. 1). Enrolment will remain open until July 2022.

#### Progression criteria

To proceed with investigating the efficacy of hypnotherapy in children with burns, at least the following criteria should be met: sufficient time to deliver the intervention in > 80% of children who commence hypnotherapy; > 50% of families with moderate to high satisfaction rate; recruitment and study completion rates > 50%; collection of > 50% of data across all time points; hypnotherapist adherence to > 50% of intervention elements in the fidelity checklist with > 50% of children; and the absence of serious side effects. The progression criteria cover components of all of the implementation outcomes (Data Synthesis, page 19, above).

## Ethical considerations

Participants will be treated according to principles of justice, beneficence, and respect for humans as stated in the “Declaration of Helsinki” following Good Clinical Practice as well as institutional, Australian, and international guidelines [57]. Hence, all children, parents, and clinicians who fulfil the inclusion criteria are fully eligible to join the study regardless of gender, ethnic background, or minority status. Following consent to participate, families will be informed verbally and in writing that they may decline participation or leave the study at any time without negative repercussions. Deviations from the approved protocol will not be allowed except when necessary to protect participants. Deviations that can influence the study conduct, outcomes, safety of participants, and procedures will require an amendment to the protocol.

### Risk management

#### Safety of the intervention

In a recent RCT conducted in the study setting, children with acute burns reported less anxiety and no adverse events with hypnotherapy [18]. In a systematic review of hypnotherapy for children’s needle-related pain and distress, no adverse events were reported [7]. Besides hypnotic suggestions, intervention components (e.g. muscle relaxation, rhythmic breathwork) and adjuncts (passive music) have been reported safe (no related adverse events) [3, 7, 42, 43]. Thus, the intervention can be considered safe with serious adverse effects unlikely.

#### Monitoring

Despite the safety of the intervention, we acknowledge the necessity of protecting children by carefully monitoring them. Adverse events will be monitored by reports from parents, children, and clinicians who review children every few days until wound healing and beyond the time of delivering hypnotherapy. Adverse events will be addressed by the hypnotherapist after each hypnotherapeutic session as specified in the intervention manual and by clinicians using a standardised management protocol of the burns department. The hypnotherapist will also consult with parents to ensure they are comfortable with the experience. The hypnotherapist will discuss children’s follow-up care options with families and provide them with the contact details of hospital personnel to whom they can raise concerns or issues. Investigators will meet weekly to monitor the study’s progress. Following the National Statement on Ethical Conduct in Human Research, events that may influence the safety of participants will be reported to the clinical health service and ethics committee within ten working days and within 48 hours if serious [57].

### Confidentiality and data management

No data will be generated by this protocol. All data collected during the proposed study, including saliva samples, will be recorded and stored in a coded form where possible. Following the Australian National Health and Medical Research Council (NHMRC) guidelines, data will be kept in a secured cabinet in a locked office and password-protected computer files. Saliva samples will be stored in a swipe card-protected laboratory freezer, to be destroyed 25 years after analysis [57]. Unless mandated by law, only the research team and the ethics committee can access participants’ records. Quantitative data may be used in future projects that are an extension of this project or by others in the field if pre-specified ethical criteria are fulfilled for accessing the data. Study results will be published in a peer-reviewed medical journal and presented at conferences. Investigators will ensure that all participants are not identifiable in related publications and will send them a summary of the research findings.

## Discussion

Evidence supports the effectiveness of hypnotherapy for procedural pain and anxiety in children with potential superiority to standard medical care, distraction techniques, and control conditions [7, 12, 13]. Despite the lack of data in children, hypnotherapy may be beneficial in treating itch by inducing nociception and relaxation given the agonist interaction between itch and distress (e.g. pain, early post-traumatic stress symptoms) [11]. Hypnotherapy may offer additional advantages such as enabling children to treat their itch and pain using self-hypnosis as well as enhancing healing, perceived self-efficacy, and coping skills [6, 8, 11]. Post-hypnotic suggestions may also be beneficial in promoting comfort, compliance, and distress relief in future procedures [8]. Yet, data on hypnotherapy for procedural pain, state anxiety, and itch are limited in children with burns.

The first known RCT on hypnotherapy in paediatric burns was limited by the absence of suggestibility screening as well as the lack of measurements of pain unpleasantness and itch outcomes [6, 18]. The absence of significant pain outcomes may have been due to the low levels of baseline pain (lower levels of baseline pain are linked to reduced pain outcomes) [58]. The study found evidence of anxiety reduction, although this was a secondary outcome with the study not powered to detect anxiety effects. An earlier RCT purported to use hypnotherapy for procedural pain and distress in children with burns also failed to identify benefits for pain outcomes; however, imagery was delivered rather than hypnotherapy [59]. Although both hypnotherapy and imagery involve imagination, they differ in the degree of absorption, neurophysiologic changes, and outcomes (e.g. hypnotherapy is accompanied by more physiological changes and pain reduction) [8, 60]. The proposed study is intended to address the lack of data on the feasibility and acceptability of hypnotherapy for procedural pain, state anxiety, and itch following screening for hypnotic suggestibility in an acute paediatric burn setting with potential barriers to the intervention’s applicability [14].

Tailoring treatment is paramount in paediatric burns given potential barriers related to the setting, clinicians, and parents as well as the unique biopsychosocial impact of burns on children’s attitude, adherence, treatment delivery, and outcomes [2, 15, 17]. Yet, pharmacological agents and distraction techniques currently used in paediatric burns lack tailoring [14, 15]. Effective tailored adjunct non-pharmacological interventions such as hypnotherapy may optimise the health care of children with burns by further reducing the already low pain reported during dressing changes and addressing state anxiety and itch, which have received less attention. Hypnotherapy can be tailored to varied settings and populations with diverse characteristics and backgrounds, which can facilitate integration into medical interventions for optimal delivery [14].

In the proposed study, hypnotherapy will be tailored to address child-related factors influencing pain and distress and keep pace with children’s changing conditions and perceptions on hypnosis, distress, and itch [8]. The intervention will be adapted to apparent child-related affective, cognitive, and sensory pain components (situational determinants) as well as symptoms of distress such as fear, discomfort, and withdrawal. Contextual and social factors of pain and distress and their interaction with internal factors will also be addressed during the intervention. For instance, the short time available to prepare children emotionally for repeated medical procedures and the duration of the hypnotic suggestibility screening (20 minutes) will make it impractical to conduct hypnotherapy following the screening. Thus, during the first dressing change, if children have a medium to high suggestibility score, the hypnotherapeutic session will be shortened to therapeutic suggestions following the standard hypnotic induction included in the hypnotic suggestibility test. Factors related to parents including misconceptions will be addressed during the pre-hypnosis interview. In addition, child-related factors that can influence hypnotic responding (e.g. attitude towards hypnosis, therapy expectations, and perceived self-efficacy) will be assessed alongside hypnotic suggestibility and addressed to promote positive outcomes [32]. However, tailoring can be challenging due to difficulties in replicating the intervention, evaluating individualised outcomes, and determining characteristics to address [61].

In the proposed study, hypnotherapy will be tailored for optimal therapy outcomes in a process that seeks to better understand potential barriers to tailoring. The use of a treatment manual describing core elements and potential adaptations will help facilitate the replication of hypnotherapy [61]. Plus, children with low hypnotic suggestibility scores will be excluded, which is anticipated to reduce variation in outcomes [61]. Characteristics that are relevant in tailoring were determined by searching literature on factors that can influence hypnotherapy outcomes, including factors of pain and hypnotic responding [61]. The collection of information on children’s characteristics, preferences, and child-related factors in the pre-hypnosis interview and suggestibility screening will inform tailoring the intervention [8]. For instance, using the collected information, the hypnotherapist will use preferred elements and likes; exclude distressing terms; address anticipatory anxiety, misconceptions, fears (e.g. towards hypnosis, pain, and the medical setting), and lack of self-efficacy; and enhance therapy expectations [8]. Fidelity measures assessing adherence to the intervention manual will inform future studies regarding factors related to the hypnotherapist (role and skills).

### Study strengths and limitations

The study will enhance knowledge about the safety of hypnotherapy by assessing the severity, timing, and duration of adverse events. The study is to our knowledge the first to assess the feasibility of hypnotic suggestibility screening in paediatric burns. Screening participants for hypnotic suggestibility may reduce participant-related confounding factors, prevent exposing children to an unnecessary burden of hypnosis, and increase hypnotherapy effects by excluding those who are unlikely to obtain pain relief with hypnotherapy. Plus, the use of an innovative mixed-methods design integrating multiple sources and data collection methods as informed by implementation frameworks can assist in identifying common barriers to implementation across settings. Yet, the success (or otherwise) of implementing the hypnotherapy intervention and evaluation procedures may be related to the local context and may not generalise to other settings. The generalisability of the study may also be limited by the exclusion of patients who require general anaesthesia for their first dressing change in the operating room and those with low hypnotic suggestibility as well as the small sample size. Excluding children with low hypnotic suggestibility scores may also lead to selection bias but is unlikely to hinder the interpretation of results considering the small rate of low suggestibility in the population (15%).

Other limitations include the lack of a control group and validation of pain unpleasantness numeric rating scale in younger children, although no other measures are available for this age group [52]. The Stanford Hypnotic Scale for Children used to measure hypnotic suggestibility is limited by the lack of validity testing with Australian children and the lack of psychometric data except that obtained during the scale development [62]. Yet, in the absence of other brief scales validated for children, the scale will be used due to the short length and adaptability to children’s preferences and age.

## Conclusion

The proposed study is the first step towards further research investigating hypnotherapy in an acute burn setting with the ultimate aim of wider clinical implementation. This study and planned future trials should guide the use of age-appropriate hypnotic suggestibility and pain measurement tools and processes that can be feasibly implemented by specialised pain management teams. The study should also guide the use of tailored hypnotic treatments in children with burns.

## Supplementary Information

**Additional file 1.** CONSORT 2010 checklist of information to include when reporting a pilot or feasibility randomized trial in a journal or conference abstract. STROBE Statement—checklist of items that should be included in reports of observational studies. SPIRIT 2013 Checklist: Recommended items to address in a clinical trial protocol and related documents.

**Additional file 2.** Intervention guidelines.

**Additional file 3.** Psychometrics and characteristics of self-report and proxy-report outcome measurement tools.

**Additional file 4.** Application of mixed method data to assess framework outcomes.

## Data Availability

Not applicable.

## Abbreviations

RCT: Randomised controlled trial
QCH: Queensland Children’s Hospital
SPIRIT: Standard Protocol Items Recommendations for Interventional Trials
StaRI: Standards for Reporting Implementation Studies initiative
CONSORT: Consolidated Standards of Reporting Trials
STROBE: Strengthening the Reporting of Observational Studies in Epidemiology
TIDieR: Template for Intervention Description and Replication
TFA: The Theoretical Framework of Acceptability
RE-AIM: Reach, Efficacy/Effectiveness, Adoption, Implementation, and Maintenance
SHCS: The Stanford Hypnotic Scale for Children
TBSA: Total body surface area
FPS-R: The Revised Faces Pain Scale
NRS: Numeric Rating Scale
VAS: Visual analogue scale
HREC: Human Research Ethical Committee
NHMRC: National Health and Medical Research Council
