# Supplementary material for: Hypnotherapy for procedural pain, itch, and state anxiety in children with acute burns: a feasibility and acceptability study protocol
Source: Pilot Feasibility Stud. 2022 Mar 9;8:58. doi: 10.1186/s40814-022-01017-z (PMC8905723; doi:10.1186/s40814-022-01017-z)
Supplement: Supplementary file 2 — Additional file 2. Intervention guidelines. [file 40814_2022_1017_MOESM2_ESM.docx]

# SUPPLEMENTARY FILE 2: INTERVENTION GUIDELINES

Table A. Guide for the pre-hypnosis interview

| **Core Elements** | | **Goals** |
| --- | --- | --- |
| **Elements to Use** | **Elements to Avoid** |  |
| - A permissive style that emphasises the importance of children’s role and active engagement for the success of hypnotherapy [1]. - Empowering, child-centred, and positive words that reflect confidence in children’s ability to change and encourage the positive use of their capacities [1]. | Authoritative terms in the form of orders or commands (e.g., *I want you to)* [1]. | To encourage children’s active participation by promoting perceived self-efficacy, sense of control and positive coping [1]. |
| Distraction of children involving directing the attention to soothing sensations and comforting sensory inputs (e.g., coolness of the water, breath), a short story or play [1]. | - Authoritative terms or terms that can trigger unpleasant feelings such as burn-related words [1]. - Personal pronouns in describing discomfort (e.g., say the discomfort instead of your discomfort) [1]. - Pain promoting language (e.g., you must be upset, it’s ok to feel bad) [1]. | To promote shift of attention and dissociation from the painful stimulus and the injury [1]. |
| Collection of information on child-related factors that can influence pain and hypnotic responding via:   - Semi-structured interview on views/beliefs towards hypnotherapy. - Ratings of perceived self-efficacy and expectations of pain relief with hypnotherapy (*How helpful do you think hypnotherapy will be for making your pain feel better?*) on 11- point Likert scales [1]. - Ratings of anticipatory anxiety regarding the procedure on a Visual Analog Scale based on Children’s Post-Operative Pain Predictors Model [1,2]. - Assessment of pain using the Revised Faces Pain Scale and questions on pain-related perception (e.g., shape, colour), sensation (e.g., stabbing, burning) and feelings (e.g., how it makes you feel), based on Rainbow Babies & Children’s Hospital General Academic Pediatrics Imagery/Discomfort Questionnaire [1]. | Neglecting children’s autonomy by obtaining information from their parents even when children are able to provide the information [1].   - Emphasis on terms that can trigger unpleasant emotions [1]. | - To inform addressing child-related factors that influence pain and hypnotic responding. - To promote children’s autonomy [1]. |
| Collection of information on children’s preferences, pleasant life experiences, imagination type/capacities, favourite music type and what they would like to imagine during hypnotherapy as well as fears, phobias and dislikes, using elements of Rainbow Babies & Children’s Hospital General Academic Pediatrics Imagery/Discomfort Questionnaire [1]. |  | - To inform tailoring of the hypnotherapy session to children’s characteristics and preferences [1]. - To avoid terms that can trigger anxiety reactions during the session [1]. - To promote children’s autonomy [1]. |
| - Explaining to children the steps that will be taken to reduce pain during hypnotherapy and answering their questions [1]. - Emphasising positive change and healing [1]. - Explaining to children that hypnotic suggestibility screening is intended to discover the best way to conduct hypnotherapy and tailor treatment rather than to evaluate their capacity to respond to suggestions [3]. | Miscommunication and talking about difficulties to attain distress relief or recovery [1]. | - To alleviate misconceptions and promote a positive attitude involving motivation and positive expectations regarding hypnotherapy [1]. - To avoid triggering feelings of failure and unmet expectations associated with lack of response to test suggestions which may lead to difficulties in responding to therapeutic suggestions [1,3]. |
| - A calm and positive attitude that inspires trust, care and respect for children and parents [1]. - Speaking to children and responding to their pain in a caring, empathetic and professional way [1]. | - Display of tension or distress to children [1]. - Inconsistent responses [1]. | To build rapport and trust [1]. |
| Explaining to children what is happening in their body and how pain works as a signal of injury with acknowledgement of their pain [1]. | - Describing pain as an enemy or invader [1]. - Using the language of war for pain relief (e.g., defeating pain) [1]. - Denial or minimising of children’s pain experience [1]. | To alleviate pain-related fears and misconceptions [1]. |
| Describing the intervention as hypnotherapy and hypnotic suggestibility as hypnotisability to children and parents [1,4]. | Describing the intervention using terms that do not refer to hypnotherapy such as imagery. | To explain the intervention while establishing rapport and a hypnotic context to promote hypnotic responding [1,4]. |
| - Cooperation with parents in conducting the semi-structured interview with respect of children’s autonomy [1]. - Informing parents about hypnotherapy, adequately explaining the process and inviting them to ask questions [1]. - Encouraging parental presence unless they chose not to be present [5]. - Explaining to parents their role in children’s medical care as partners who should respond to children’s pain and behaviour by promoting healthy coping [5]. - Highlighting children’s inner resources, abilities and autonomy in hypnotherapy [1]. | - Over-involvement from parents such as answering questions related to children who are able to answer [1]. - Asking parents to leave [5]. - Eliciting parental distress (blaming, coercion or restrains) [5]. | To alleviate misconceptions; promote active and positive parental involvement in child-care and a positive attitude that supports children’s therapeutic progress [1]. |

Table B. Brief description of music medicine as an adjunct component to hypnotherapy

| **Description** | **Goals** |
| --- | --- |
| - Provide music starting in a tranquil rhythm then gradually becoming more reminiscent following deepening until emergence from hypnosis [6]. - Deliver hypnotic suggestions to promote relaxation and pleasant feelings through music (e.g., *the music that you are listening to will help you to relax m*ore) [1].   N.B. Music pieces are selected from a range of sources as guided by a music therapist based on the evoked emotion and children’s preferences (e.g., soundtracks from their favourite movie if applicable and favourite music type as informed by the pre-hypnosis phase). | - To induce relaxation via tranquil music, and pleasant feelings via reminiscent cheerful music [6]. - To promote passive distraction, absorption in hypnosis, hypnotic responsiveness and distress relief through music [6]. - To reduce procedural pain and state anxiety [6,7]. |

Table C. Addressing and assessing factors of hypnotic responding in the study

| **Factors** | **Strategies to Address Factors** | | **Assessment of Factors** | |
| --- | --- | --- | --- | --- |
|  | **Description** | **Time-Point** | **Description** | **Time-Point** |
| Parental influence: attitude and involvement [4] | - Inform parents about hypnotherapy and explain their role in children’s medical care to promote positive responding and coping [1,5]. - Encourage positive parental involvement while respecting children’s autonomy [1]. | During the pre-hypnosis interview, before the 1^st^ dressing change. | Assessment of parental attitude by willingness to take part in the study and views/beliefs towards hypnotherapy. | During the pre-hypnosis interview before the 1^st^ dressing change |
| Contextual factors: potential disruptions and short time-frame [4] | Shorten the hypnotherapy session to therapeutic suggestions following the SHCS-CHILD test that involves a standard induction and test suggestions [8]. | During the hypnotherapy session, at the 1^st^ dressing change. | Assessment of feasibility that provides information on contextual barriers and facilitators to the delivery of the intervention. | Following data collection, during analysis of intervention pre-recordings. |
|  | - Ensure a calm space. - Use music to reduce external noise influence. | During the hypnotherapy session, at each dressing change. |  |  |
| Hypnotic context [4] | Establish the hypnotic context by describing the intervention as hypnotherapy [1]. | During the pre-hypnosis, interview, before the 1^st^ dressing change. | Assessment of the fidelity in delivering the intervention. | Following data collection, during the analysis of intervention pre-recordings. |
| Hypnotic aptitude: absorption, dissociation, suggestibility, and imaginative abilities [4]. | Screen out participants with low hypnotic suggestibility [9]. | During the pre-hypnosis suggestibility screening at the 1^st^ dressing change. | - Hypnotic suggestibility screening (SHCS: CHILD) [8] - Assessment of involuntariness experienced during response to hypnotic suggestions [10] | During the pre-hypnosis suggestibility screening, at the 1^st^ dressing change |
|  | Encourage visualisation and use of imaginative abilities [1]. | During the hypnotherapy session, at each dressing change. | Assessment of imagination type and abilities using elements of Rainbow Babies & Children’s Hospital General Academic Pediatrics Imagery/Discomfort Questionnaire [1] | During the pre-hypnosis interview before the 1^st^ dressing change. |
| Children’s attitude towards hypnotherapy: expectations, motivation, and views [4]. | Promote a positive attitude towards hypnotherapy (including positive expectations and motivation) and dispel misconceptions [1]. | During the pre-hypnosis interview, before the 1^st^ dressing change. | - Assessment of attitude by willingness to join the study and views/beliefs towards hypnotherapy. - Assessment of therapy expectations on a 0-10 Likert scale. | During the pre-hypnosis interview before the 1^st^ dressing change. |
| Rapport with the hypnotherapist [4] | Build trust and a positive rapport by promoting empathy, confidence and establishing a therapeutic relationship [1]. | During the pre-hypnosis interview, before the 1^st^ dressing change. | Assessment of the fidelity in delivering the intervention | Following data collection, during the analysis of intervention pre-recordings. |
| SHCS-CHILD: The Stanford Hypnotic Clinical Scale for children. | | | | |

Table D. Addressing and assessing psychosocial factors of pain and distress

| **Psychological (cognitive, behavioural, emotional) Child-Related Factors** | | **Strategies to Address Factors** | | **Assessment of Factors** | |
| --- | --- | --- | --- | --- | --- |
| **Occurrence** | **Description** | **Description** | **Time-Point** | **Description** | **Time-Point** |
| Situational | Cognitive processing of pain: perceptions, related thoughts, understanding of cause and prognosis, expectations (e.g., continuing pain) [11,12]. | - Promote positive expectations of pain treatment [1]. - Promote acceptance and understanding of the important role of pain as a signal of injury [1]. | During the pre-hypnosis interview before the 1^st^ dressing change. | N/A  The occurrence of situational factors during the intervention limits the practicality of assessing them | N/A |
|  |  | Address potential fears and negative perceptions related to pain by promoting positive views and perceptions [1]. | - During the pre-hypnosis interview before the 1^st^ dressing change. - During hypnotherapy sessions. |  |  |
|  | Coping: engagement (catastrophizing, awareness, reframing pain-related emotions and thoughts) to disengagement (avoidance, distraction) in painful stimulus and related thoughts or emotions [11,12]. | Promote positive coping strategies by encouraging compliance, distraction, mindfulness, acceptance and modulation of pain-related emotions and thoughts (reframing) [1]. | - During the pre-hypnosis interview before the 1^st^ dressing change. - During hypnotherapy sessions. |  |  |
|  | Attention: involuntary attention in response to pain followed by coping and voluntary attention [13]. | Shift the attention away from the painful stimulus [1]. |  |  |  |
|  | Perceived control: sense of control, resilience or hopelessness [11,12] . | Empowering children and reinforcing their perceived sense of control over bodily processes [1] . | - During the pre-hypnosis interview, before the 1^st^ dressing change. - During the hypnotherapy session. |  |  |
|  | Emotional Response: frustration, anger, distress (anxiety, fear) [11]. | Address distress and unpleasant emotions while promoting relaxation and pleasant feelings [1] . |  |  |  |
| Predisposing | Age and cognitive level [11] | Word the intervention according to children’s cognitive level and language capacity [1]. | - During the pre-hypnosis interview before the 1^st^ dressing change. - During hypnotherapy sessions at each dressing change | - Assessment of cognitive deficits by clinicians’ psychosocial screening, or medical records. | During recruitment or screening, before the 1^st^ dressing change. |
|  |  | Exclude children with cognitive deficits | During recruitment or screening before the 1^st^ dressing change |  |  |
|  | - Mental health: psychopathology (e.g., depression) [11,12] - Personality variables (e.g., fearful, shy) [12] | Screen out children with severe psychopathology that can affect their response to hypnotic suggestions. |  | - Identification of patients with severe psychopathology through psychosocial screening (collection of information on previously diagnosed psychiatric disorder) by clinicians |  |
|  | Cognitive content: beliefs regarding pain, self-efficacy and disability [12] | - Alleviate misbeliefs on pain by describing it as a signal of injury rather than an enemy [1]. - Address misbeliefs on the medical setting and resistance to painful procedures [1]. - Promote acceptance, compliance, trust in clinicians and confidence in internal resources [1]. | - During the pre-hypnosis interview before the 1^st^ dressing change. - During hypnotherapy sessions. | - Assessing perceived self-efficacy on a 0-10 Likert scale - Assessing misbeliefs in a semi-structured interview | During the pre-hypnosis interview, before the 1^st^ dressing change. |
| **Contextual/Social factors** | | **Hypnotic Strategies to Address Factors** | | **Assessment of Factors** | |
| **Occurrence** | **Description** | **Description** | **Time-Point** | **Description** | **Time-Point** |
| Situational | Nature of the setting: hospitalisation and the unfamiliarity of the medical setting [14,15]. | Address negative feelings (e.g., fear), attitudes and perceptions regarding hospitalisation and the hospital setting [1]. | - During the pre-hypnosis interview before the 1^st^ dressing change. - During hypnotherapy sessions. | Assessing fears towards the setting, treatment procedures and pain. | During the pre-hypnosis interview, before the 1^st^ dressing change. |
|  | Clinicians:  attitude and behaviour that promote pain or coping; assistance or interruption in therapy (cooperation and teamwork) [12]. | Use a cooperative, respectful approach with clinicians that promotes teamwork. | - During the pre-hypnosis interview before the 1^st^ dressing change. - During hypnotherapy sessions | Assessing clinicians’ attitude by their willingness to take part in the study | During recruitment |
|  | Parental influence:  distress, attitudes and actions (protective, therapeutic) [5,11,12]. | - Promote parental support, as well as positive attitude and involvement [1,5]. - Avoid the occurrence of separation anxiety due to absence of parents [5]. | - During the pre-hypnosis interview, before the 1^st^ dressing change. - During hypnotherapy sessions. | Assessing parental attitude by the willingness to take part in the study and views towards the intervention | - During recruitment - During the pre-hypnosis interview before the 1^st^ change |
|  | Interpersonal relationships: siblings, peer [11]. | Not addressed by the intervention | | N/A | N/A |
| Predisposing | - Family and cultural learning [11] - Prior pain and hospital experiences (e.g., negative conditioning) [12] | Dispel misbeliefs and fears; promote positive conditioning; treat pain and anxiety from an early phase of treatment (1^st^ dressing change) to reduce conditioning factors. | - During the pre-hypnosis interview before the 1^st^ dressing change. - During the 1^st^ hypnotherapy session, at the 1^st^ dressing change. | Assessing negative conditioning and misbeliefs regarding the medical setting | During the pre-hypnosis interview, before the 1^st^ dressing change. |
| N/A: not available. | | | | | |

Table E. Fidelity checklist for the delivery of the intervention

| **Phase** | **Components** | **Delivery Assessment** | | | |
| --- | --- | --- | --- | --- | --- |
| Pre-hypnosis interview | Collection of information on | Views/beliefs | | | Yes □ No □ |
|  |  | Perceived self-efficacy | | | Yes □ No □ |
|  |  | Positive therapy expectations | | | Yes □ No □ |
|  |  | Preferences (e.g., preferred music type) and likes | | | Yes □ No □ |
|  |  | Dislikes, fears | | | Yes □ No □ |
|  |  | Language skills | | | Yes □ No □ |
|  |  | Imagination type and capacities | | | Yes □ No □ |
|  |  | Pain-related beliefs, perception, sensations and feelings. | | | Yes □ No □ |
|  | Provision of information on | Hypnotherapy and children’s role | | | Yes □ No □ |
|  |  | Parents’ role | | | Yes □ No □ |
|  |  | Pain, provided treatment and the setting | | | Yes □ No □ |
|  | Pre-hypnosis talk and the use of language | Addressing misconceptions | | | Yes □ No □ |
|  |  | Promoting positive therapy expectations | | | Yes □ No □ |
|  |  | Empowering and promoting perceived self-efficacy | | | Yes □ No □ |
|  |  | Reframing | | | Yes □ No □ |
|  |  | Patient-centred | | | Yes □ No □ |
|  |  | Empathising on relief from pain, anxiety and itch | | | Yes □ No □ |
| Hypnotic suggestibility screening | Induction | Delivery as described in the SHCS-CHILD manual | | | Yes □ No □ |
|  | Test items | Delivery as described in the SHCS-CHILD manual | | | Yes □ No □ |
| Involuntariness measures | Questions on involuntariness | Asking the child to give an ideomotor response to signal experienced involuntariness | | | Yes □ No □ |
|  |  | Asking about experienced involuntariness after each test suggestion* | | | Yes □ No □ |
|  |  | Taking note of given ideomotor response | | | Yes □ No □ |
| Induction** | Positioning | Yes □ No □ | | | |
|  | Eye fixation | Yes (duration: up to one minute) □ No □ | | | |
|  | Abbreviated muscle relaxation | Yes (duration: up to one minute) □ No □ | | | |
|  | Deliberate faster breathwork | Yes (duration: few minutes) □ No □ | | | |
|  | Relaxation and pleasant imagery suggestions | Yes □ No □ | | | |
| Deepening** | Deepening suggestions | Yes □ No □ | | | |
|  | Testing hypnotic depth | Was the hypnotic depth tested? | Yes □ No □ | | |
|  |  | Which suggestions were given to test the hypnotic depth? | - Eyelid catalepsy | □ | N.B. At least two types of test suggestions should be given to test the hypnotic depth. |
|  |  |  | - Limb catalepsy | □ |  |
|  |  |  | - Arm heaviness | □ |  |
|  |  | Did the child give positive behavioural response to the given suggestions? | - Eyelid catalepsy | □ | N.B. The child should respond positively to at least two test suggestions before proceeding to therapeutic suggestions. |
|  |  |  | - Limb catalepsy | □ |  |
|  |  |  | - Arm heaviness | □ |  |
| Therapeutic suggestions | Hypnotic suggestions | Which suggestions for glove anaesthesia and hypnotic analgesia were given? | - Direct ideosensory | □ | N.B. At least two types of hypnotic suggestions should be given. |
|  |  |  | - Age-regression | □ |  |
|  |  |  | - Pleasant imagery | □ |  |
|  |  |  | - Reframing metaphor | □ |  |
|  |  | Was the child’s response to suggestions tested by an ideomotor response? (lifting of the finger) | - Direct ideosensory | □ | N.B. The child should be responsive to at least two types of suggestions |
|  |  |  | - Age-regression | □ |  |
|  |  |  | - Pleasant imagery | □ |  |
|  |  |  | - Reframing metaphor | □ |  |
|  |  | If the child was not responsive to at least two types of suggestions, were deepening suggestions given, followed by therapeutic suggestions? | - Deepening suggestion | □ |  |
|  |  |  | - Direct ideosensory | □ |  |
|  |  |  | - Age-regression | □ |  |
|  |  |  | - Pleasant imagery | □ |  |
|  |  |  | - Reframing metaphor | □ |  |
|  |  | Was the child’s response to suggestions tested by an ideomotor response (lifting of the finger)? | - Direct ideosensory | □ |  |
|  |  |  | - Age-regression | □ |  |
|  |  |  | - Pleasant imagery | □ |  |
|  |  |  | - Reframing metaphor | □ |  |
| Post-Hypnotic Suggestions | Anchoring | Was an anchoring technique used with the child? | Yes □ No □ | | |
|  |  | Was the child’s response to anchoring tested by ideomotor response (lifting of the finger)? | Yes □ No □ | | |
|  |  | If the child was not responsive to the 1^st^ anchoring technique, was another anchoring technique used and tested? | Yes □ No □ | | |
|  | Future progression | Was the child given suggestions of pleasant future imagery? | For itch, pain and anxiety relief | □ |  |
|  |  |  | For adherence with future burn-care procedures | □ |  |
|  |  | Was the child’s response to suggestions tested by an ideomotor response (lifting of the finger)? | Yes □ No □ | | N.B. The child should be asked to give an ideomotor signal in response to future-related suggestions to indicate that they perceive the future as described in the given suggestions. |
|  | Self-hypnosis | Were suggestions for self-hypnosis given to the child? | Yes □ No □ | | |
| Emergence from hypnosis | Counter-suggestions | Was the child given suggestions to emerge from hypnosis? | Yes □ No □ | | |
|  |  | Was the child asked to give a sign if their focus is back to the external setting? | Yes □ No □ | | |
|  |  | If the child needed more time, were more counter suggestions given and tested? | Yes □ No □ | | |
|  |  | When the child was back to the normal waking state, were they asked to take the time to open their eyes and go back gradually to a seated position? | Yes □ No □ | | |
| Post-Hypnosis | Post-hypnotic talk | Was the child approached after the session? | Yes □ No □ | | |
|  |  | Was the child assessed for adverse reactions? | Yes □ No □ | | |
|  |  | Were adverse reactions reported? | Yes □ No □ | | |
|  |  | Were adverse reactions adequately addressed and minimised, if any***? | Yes □ No □ | | Adverse reactions should be assessed after being addressed to make sure they are minimised. |
|  |  | Were the inquiries of the child answered, if any? | Yes □ No □ | | |
|  | Self-hypnosis (1^st^ visit) | Was the child given a pre-recording of self-hypnosis? | Yes □ No □ | | |
|  |  | Was the child invited to ask questions and given needed explanations of self-hypnosis if needed | Yes □ No □ | | |
| Music medicine accompanying the session | Tranquil and cheerful music | Was the child’s preferred music type provided? | Yes □ No □ | | |
|  |  | Was the music volume adjusted to the child’s preference? | Yes □ No □ | | |
|  |  | Was the music more tranquil during induction then more reminiscent during emergence? | Yes □ No □ | | |
| * Experienced involuntariness during responses to SHCS-C items will be assessed using age-appropriate question reworded according to children’s age.  **Induction and deepening are omitted during the first visit and replaced by standard induction in the SHCS-C. *** Adverse reactions that can occur during the hypnotherapy session should also be addressed and minimised. | | | | | |

Table F. Description of possible adaptations of the intervention

| **Phase** | **Adaptation** | | **Goal of the Adaptation** |
| --- | --- | --- | --- |
|  | **Elements to Use** | **Elements to Avoid** |  |
| Pre-hypnosis interview | Use child-oriented language according to children’s age and apparent language capacities (e.g., child-familiar terms; easier, simpler words and shorter sentences with younger children) [1]. | Use difficult language without consideration of children’s age (e.g., complex or hard to understand words) [1] . | To tailor the talk to children’s language, cognitive and communication capacities [1]. |
|  | Offer assistance and support to anxious children using empowering words while teaching them positive coping for stress management such as mindfulness with breath awareness and distraction with pleasant imagery [1]. | Use terms that can elicit anxiety [1]. | - To alleviate distress and unpleasant emotions by inducing relaxation and pleasant feelings [1]. - To promote positive coping [1]. |
|  | Offer additional talk dispelling present misconceptions and fears towards hypnotherapy, pain and the setting while emphasising pain relief and healing. | - Mentioning difficulties and barriers to attain pain relief [1]. - Using negative terms that elicit fear and distress towards pain, hypnotherapy or the setting [1]. | To address negative views and attitude towards hypnotherapy and therapy expectations [1]. |
|  | The hypnotherapist will consult with parents to ensure they are comfortable with the experience [1,5]. | Eliciting parental distress (e.g., blaming) [5]. | To reduce parental distress [1,5] |
|  | Additional empowering talk that emphasises on ability to change and addresses misbeliefs regarding the ability to engage in hypnotherapy [1]. | Mentioning difficulties and barriers to attain pain relief and negative and terms reflecting children’s lack of ability. | To address low self-confidence and sense of control. |
| Induction | Enable children to slow down the rhythm of breathwork | | To adjust children’s breathing rhythm to their preferences and level of comfort [1]. |
|  | - Use shorter and more simple inductions that include easier words with younger children and those with less advanced cognitive development and language capacities [1,8]. - Use more complex and longer inductions that include less simplistic words with older children and those who have more advanced cognitive development and language capacities [8]. | | To tailor the induction to children’s age, language capacities and cognitive development [8]. |
|  | - Use an active fantasy induction with younger children below the age of six that allows them to keep their eyes open [1,8]. - Use an induction with eyes closed in older children [8]. | | To adapt the session to children’s preferences and comfort as well as prevent resistance towards hypnosis [8].  N.B. Children below the age of six may resist typical eye closure inductions and can be in favour of an active fantasy induction [1,8]. |
| Deepening | Use a different deepening technique if the child has an unpleasant experience or feelings associated with stairs [1] . | | To prevent unpleasant feelings and anxiety reactions [1]. |
|  | If the child does not respond to suggestions for deepening:   - avoid triggering feelings of failure; - give more suggestions involving breathing techniques until a positive response is established [1] . | | To promote responsiveness to suggestions [1]. |
| Therapeutic suggestions | - Adapt the content of indirect suggestions to children’s preferences by including their favourite elements based on their imagery type (e.g., auditory, visual, tactile) [1]. - Avoid negative words and terms that can trigger distress (as reported in the pre-hypnosis interview) or that are related to burns [1]. | | - To make the session more appealing, absorbing and relevant to children [1]. - To avoid triggering fear and distress [1]. |
|  | - Word the session according to children’s age, language capacities and cognitive development as informed by the pre-hypnosis interview and hypnotic suggestibility testing [1]. - Offer additional assistance and reword hypnotic suggestions with younger children and those with less advanced language capacities and cognitive development (e.g., easier words and shorter sentences) [1]. | | - To adapt the session to children’s age, language capacities and cognitive development [1]. - To prevent failure of therapy by ensuring that children understand the used terms to be able to respond to suggestions and engage in the hypnotic process [1] . |
|  | Use suggestion types that the child is responsive to as informed by hypnotic suggestibility screening [8]. | | To promote responsiveness to suggestions [1,8]. |
|  | If the child does not give an ideomotor sign of positive response (finger lifting) to suggestions, give more suggestions until establishing positive response [1]. | | To ensure a positive response to suggestions for pain and anxiety relief [1]. |
|  | Use metaphors of objects representing unpleasant symptoms and emotions that can be overcome, reduced or substituted to dissociate the child from unpleasant emotions and replace them with pleasant ones (e.g., fear can be described as a darkness that can be released or transformed into light) [1]. | | - To alleviate apparent symptoms of distress such as fear, anxiety and any unpleasant emotion related to the setting, the injury and/or the experienced pain and distress [1]. - To promote positive change and sense of control [1]. |
|  | Give additional suggestions that promote children’s sense of control, perceived self-efficacy and positive therapy expectations [1] . | | To address children’s lack of perceived self-efficacy and negative therapy expectations as assessed by the pre-hypnosis interview [1] . |
|  | - Ask children to describe their experience while showing support and acceptance [1]. - Use calming and protective suggestions for children to change perceptions, and to be more positive about the experience [1]. - Distract children away from the memory if not resolved and recommend professional psychologic support after the session [1]. - Use soothing and confident voice, and slow movement as opposed to sudden, quick movements [1]. | | To address spontaneous regression to an unpleasant memory [1] . |
| Post-hypnosis | Debrief children and address adverse reactions [1].* | | To ensure the safety of child participants [1]. |
| *Adverse reactions that can occur during hypnotherapy should also be addressed and minimised during the session.  N.B. The intervention will be adapted to children’s talkativeness and activity by suggesting relaxation and decreased movement as well as agreeing upon a “waking up” signal that is reassuring to many children [1]. | | | |

Table G. Medications and dosages commonly used for dressing changes in the study setting

| **Medication** | **Dosage** | **Route of administration** |
| --- | --- | --- |
| Oxycodone | 0.1 mg/kg | Orally |
| Paracetamol | 150 mg/kg |  |
| Ibuprofen | 10mg/kg |  |

**REFERENCES**

1. Kohen DP, Olness K. Hypnosis and Hypnotherapy with Children: Routledge; 2012.

2. Palermo TM, Drotar D. Prediction of children's postoperative pain: the role of presurgical expectations and anticipatory emotions. J Pediatr Psychol. 1996;21(5):683-98. doi: 10.1093/jpepsy/21.5.683 [published Online First: 1996/10/01]

3. Frankel FH, Orne MT. Hypnotizability and phobic behavior. Arch Gen Psychiatry. 1976;33(10):1259-61. doi: 10.1001/archpsyc.1976.01770100121012

4. Jensen MP, Adachi T, Tomé-Pires C, Lee J, Osman ZJ, Miró J. Mechanisms of hypnosis: toward the development of a biopsychosocial model. Int J Clin Exp Hypn. 2015;63(1):34-75. doi: 10.1080/00207144.2014.961875 [published Online First: 2014/11/05]

5. Brown EA, De Young A, Kimble R, Kenardy J. Review of a parent’s influence on pediatric procedural distress and recovery. Clin Child Fam Psychol Rev. 2018;21(2):224-45. doi: 10.1007/s10567-017-0252-3

6. Klassen JA, Liang Y, Tjosvold L, Klassen TP, Hartling L. Music for pain and anxiety in children undergoing medical procedures: a systematic review of randomized controlled trials. Ambul Pediatr. 2008;8(2):117-28. doi: 10.1016/j.ambp.2007.12.005 [published Online First: 2008/03/22]

7. Birnie KA, Noel M, Parker JA, Chambers CT, Uman LS, Kisely SR, et al. Systematic Review and meta-analysis of distraction and hypnosis for needle-related pain and distress in children and adolescents. J Pediatr Psychol. 2014;39(8):783-808. doi: 10.1093/jpepsy/jsu029 [published Online First: 2014/06/04]

8. Morgan AH, Hilgard JR. The Stanford Hypnotic Clinical Scale for children. Am J Clin Hypn. 1978;21(2-3):148-69. doi: 10.1080/00029157.1978.10403969 [published Online First: 1978/10/01]

9. Lynn SJ, Shindler K. The role of hypnotizability assessment in treatment. Am J Clin Hypn. 2002;44(3-4):185-97. doi: 10.1080/00029157.2002.10403479 [published Online First: 2002/01/22]

10. Bowers KS. Do the Stanford scales tap the “classic suggestion effect”? Int J Clin Exp Hypn. 1981;29(1):42-53. doi: 10.1080/00207148108409142

11. Turk DC, Melzack R. Assessment of pain in infants, children, and adolescents. Handbook of pain assessment: Guilford Press; 2011.

12. Racine NM, Pillai Riddell RR, Khan M, Calic M, Taddio A, Tablon P. Systematic review: predisposing, precipitating, perpetuating, and present factors predicting anticipatory distress to painful medical procedures in children. J Pediatr Psychol. 2015;41(2):159-81. doi: 10.1093/jpepsy/jsv076 [published Online First: 2015/09/05]

13. McGrath PJ, Frager G. Psychological barriers to optimal pain management in infants and children. Clin J Pain. 1996;12(2):135-41. doi: 10.1097/00002508-199606000-00009 [published Online First: 1996/06/01]

14. Yeh VM, Schnur JB, Montgomery GH. Disseminating hypnosis to health care settings: Applying the RE-AIM framework. Psychol Conscious. 2014;1(2):213-28. doi: 10.1037/cns0000012 [published Online First: 2014/10/01]

15. Nelson S, Conroy C, Logan D. The Biopsychosocial model of pain in the context of pediatric burn injuries. Eur J Pain. 2019;23(3):421-34. doi: 10.1002/ejp.1319 [published Online First: 2018/10/06]
