# Supplementary material for: Hypnotherapy for procedural pain, itch, and state anxiety in children with acute burns: a feasibility and acceptability study protocol
Source: Pilot Feasibility Stud. 2022 Mar 9;8:58. doi: 10.1186/s40814-022-01017-z (PMC8905723; doi:10.1186/s40814-022-01017-z)
Supplement: Supplementary file 3 — Additional file 3. Psychometrics and characteristics of self-report and proxy-report outcome measurement tools. [file 40814_2022_1017_MOESM3_ESM.docx]

## SUPPLEMENTARY FILE 3

Psychometrics and characteristics of self-report and proxy-report outcome measurement tools

| **Scale** | **Description** | **Psychometrics** | **Advantages** | **Age** |
| --- | --- | --- | --- | --- |
| FPS-R [1] | Self-report scale consisting of 6 gender-neutral faces allocated numerical values from 0 to 10, ranging from “no pain” portrayed by a neutral face to “very much pain” depicted by a sad but not tearful face [1]. | - Good discriminative validity: higher scores in the group with painful conditions than in the non-pain group (n = 620) [2]. - Adequate criterion validity: moderate correlation between FPS-R and FAS scores (r = 0.42 - 0.67, n = 124) with statistically significant coefficient [3]. - Good construct validity: strong correlation with parents’ scores (r = 0.84, n = 620) [2,3]. - Convergent validity: strong correlation with VAS (r = 0.93, n = 76), CAS (r = 0.84, n = 75 and n = 620; r = 0.83 - 0.90, n = 371), WBFPS (r = 0.79, n = 122), and NRS (r = 0.78, n = 175) [3]^.^ - Responsiveness: high sensitivity to pain reduction with anaesthetics, analgesia (e.g., MD = − 1.61, SD = 2.00, n = 456) and non-pharmacological interventions or painful procedures [2-4]. - High test-retest reliability (r = 0.77, repeatability coefficients = 0.53, n = 40) [2], (r = 0.76, t = − 6.35, n = 456) [4]. | - Has high feasibility: low cost (free of charge), easy use, brief duration, minimal instructions requirements [3]. - Involves numeric values with equivalent intervals to quantify pain [3]. - Has less confounding of emotional and sensory aspects of pain (less upper-end bias, e.g., 2.64% of 456 children) than other scales that show more emotional facial expressions (e.g., WBFPS) such as smiley and tearful ones [3]. - Rated as more preferred than the VAS and CAS by children and nurses [3,4]. | 4 - 17 yrs. [2,3] |
| NRSI [5] | Numeric scale with anchors ranging from 0 (no pain) to 10 (worst pain possible) [5]. | - Adequate discriminative validity: moderate correlation between intensity and unpleasantness of pain 48 to 72 hrs and 2 weeks after hospital discharge and functional disability assessed 2 weeks after hospital discharge (n = 83) [5]. - Good construct validity as shown in median scores variation following analgesia (from 6.5 [IQR 4.5, 7] before analgesia to 4 [IQR 2, 6] after, n = 202) [6]. - Good content validity as shown by differences in the median change scores between the mild and the moderate categories and between the moderate and the severe categories (n = 202) [6]. - Criterion validity established using measures of pain affect correlation with FAS (r = 0.66, n = 63; r = 0.58, n = 175), disability (r = 0.22, n = 175; r = 0.39, n = 63), and quality of life (r = 0.46, n = 63) [7]. - Good convergent validity: strong correlation with FPS-R (r = 0.78, n = 175; r = 0.93, n = 63) [7], VAS (r = 0.93, n = 202) [6] and VRSI (P < 0.01, n = 83) [5]. - Sensitivity to change: significant decrease from 48 hours to 72 hours to 2 weeks from discharge (P < 0.001, n = 83) [5], and after analgesics (P < 0.001, n = 202) [6]. - High test-retest reliability (MD = 0.2, n = 202) [6]. | - Has low cost and brief duration [5]. - Is applicable in the research setting [5]. - Is simple, familiar and easy to use (no equipment requirement) [5]. - Is easy to understand and to quantify with numbers [5]. - Avoids talk of pain [5]. | 8-18 yrs. (used in 6-16 yrs.) [5]. |
| NRSU [5] | Numeric scale with anchors ranging from 0 (not at all unpleasant/yucky/horrible) to 10 (most unpleasant/horrible/yucky) [5]. | - Established discriminant validity for both pain unpleasantness and intensity in young children: moderate correlation with NRSI (r = 0.3 - 0.7, n = 83) [5]. - Good convergent validity: strong correlation with FAS (*P* = 0.01, n = 83) and VRSU-1 (*P* = 0.02, n = 83) [5]. - Sensitivity to change in pain from 38 hours to 72 hours to 2 weeks from discharge (*P* < 0.01, n = 83) [5]. |  |  |
| VAS[8] | Self-report visual scale consisting of a 100mm horizontal line marked with anchors on a continuum ranging from the absence of anxiety at one extreme toward highest anxiety at the other extreme [8].  N.B. The patient is asked to mark the line that matches the amount of currently experienced anxiety, and a score is formed by measuring the distance from the mark to the lowest set-point [8]. | - Adequate discriminative validity: moderate correlation with pain scales such as FPS and VAS (r = 0.52 at discharge, r = 0.37 at post-operative period, n = 100) with differentiation of anxiety and pain [8]. - Adequate convergent validity: moderate correlations with other anxiety assessment tools such as the STAIC (r = 0.66, n = 100), STAIY (r = 0.67, n = 100), and m-YPAS (r = 0.67, n = 100) [8]. - Responsiveness: sensitivity to the presence of anxiety disorders and distraction [8]. - Test-retest reliability: moderate to strong at 2-hour and 1-week intervals (r = 0.53-0.87, n = 70) [8]. | - Is useful in longitudinal studies due to sensitivity to small changes over time [8]. - Requires minimal verbal expression [8]. - Is easy to use, simple and brief [8]. | 7-16  (children with sufficient cognitive and language skills to understand numbers, distance, and mark the line that matches their anxiety level) [8] |
| Itch-NRS [9] | 11-point scale (0 = absence of itch; 10 = most excruciating itch) [9] | - Content validity established as developed based on interviews with children with burn scars and their parents [9]. - Good convergent validity in children with burns: strong correlation with Itch Man Scale (r = 0.82, n = 255) [10]. - High test-retest reliability (ICC = 0.71 -0.83, n = 65) [11]. | - Is easy to use in children with hand dressings [9]. - Has age-appropriate and easy to understand content [9]. | - Psychometrics tested for ≥ 8yrs [9]. - Successful use in 3-16 yrs.: ≥ 6 yrs. rated their itch whereas parent proxy reports were used for younger children [9,10]. |
| ICC: Intra-class Correlation Coefficients; r: Pearson correlation; MD: mean difference; IQR: interquartile range; CAS: Color Analog Scale; VAS: Visual Analog Scale; NRS: Numeric Rating Scale; FPS-R: Faces Pain Scale-Revised; FPS: Faces Pain Scale; WBFPS: Wong-Baker Scale; NRSI: Numerical Rating Scale for Pain Intensity; NRSU: Numerical Rating Scale for Pain Unpleasantness; VRSU: Verbal Rating Scale for Pain Unpleasantness; FAS: Facial Affective Scale; STAIY: State Spielbergers’ questionnaires Inventory for Youth; STAIC: State-Trait Anxiety Inventory for Children; M-YPAS: modified Yale Preoperative Anxiety Scale. | | | | |

**References**

1. Hicks CL, von Baeyer CL, Spafford PA, van Korlaar I, Goodenough B. The Faces Pain Scale–Revised: toward a common metric in pediatric pain measurement. Pain. 2001;93(2):173-83. doi: 10.1016/s0304-3959(01)00314-1 [published Online First: 2001/06/28]

2. Tsze DS, von Baeyer CL, Bulloch B, Dayan PS. Validation of self-report pain scales in children. Pediatrics. 2013;132(4):e971-e9. doi: 10.1542/peds.2013-1509 [published Online First: 2013/09/04]

3. Tomlinson D, von Baeyer CL, Stinson JN, Sung L. A systematic review of faces scales for the self-report of pain intensity in children. Pediatrics. 2010;126(5):1168-98. doi: 10.1542/peds.2010-1609 [published Online First: 2010/10/06]

4. Le May S, Ballard A, Khadra C, Gouin S, Plint AC, Villeneuve E, et al. Comparison of the psychometric properties of 3 pain scales used in the pediatric emergency department: visual analogue scale, faces pain scale-revised, and colour analogue scale. Pain. 2018;159(8):1508-17. doi: 10.1097/j.pain.0000000000001236

5. Pagé MG, Katz J, Stinson J, Isaac L, Martin-Pichora AL, Campbell F. Validation of the numerical rating scale for pain intensity and unpleasantness in pediatric acute postoperative pain: sensitivity to change over time. J Pain. 2012;13(4):359-69. doi: 10.1016/j.jpain.2011.12.010 [published Online First: 2012/03/20]

6. Bailey B, Daoust R, Doyon-Trottier E, Dauphin-Pierre S, Gravel J. Validation and properties of the verbal numeric scale in children with acute pain. Pain. 2010;149(2):216-21. doi: 10.1016/j.pain.2009.12.008 [published Online First: 2010/03/02]

7. Miró J, Castarlenas E, Huguet A. Evidence for the use of a numerical rating scale to assess the intensity of pediatric pain. European Journal of Pain. 2009;13(10):1089-95. doi: 10.1016/j.ejpain.2009.07.002 [published Online First: 2009/09/04]

8. Foster RL, Park JH. An integrative review of literature examining psychometric properties of instruments measuring anxiety or fear in hospitalized children. Pain Manag Nurs. 2012;13(2):94-106. doi: 10.1016/j.pmn.2011.06.006 [published Online First: 2012/06/02]

9. Tyack Z, Ziviani J, Kimble R, Plaza A, Jones A, Cuttle L, et al. Measuring the impact of burn scarring on health-related quality of life: Development and preliminary content validation of the Brisbane Burn Scar Impact Profile (BBSIP) for children and adults. Burns. 2015;41(7):1405-19. doi: 10.1016/j.burns.2015.05.021 [published Online First: 2015/10/01]

10. Blankers K, Dankerlui N, van Loey N, Pursad M, Rode H, van Dijk M. Cross-cultural validation of the Itch Man Scale in pediatric burn survivors in a South African setting. Burns. 2019;45(3):725-31. doi: 10.1016/j.burns.2018.09.027 [published Online First: 2018/12/12]

11. Simons M, Kimble R, McPhail S, Tyack Z. The Brisbane Burn Scar Impact Profile (child and young person version) for measuring health-related quality of life in children with burn scars: a longitudinal cohort study of reliability, validity and responsiveness. Burns. 2019;45(7):1537-52. doi: 10.1016/j.burns.2019.07.012 [published Online First: 2019/08/08]
