# Supplementary material for: Hypnotherapy for procedural pain, itch, and state anxiety in children with acute burns: a feasibility and acceptability study protocol
Source: Pilot Feasibility Stud. 2022 Mar 9;8:58. doi: 10.1186/s40814-022-01017-z (PMC8905723; doi:10.1186/s40814-022-01017-z)
Supplement: Supplementary file 4 — Additional file 4. Application of mixed method data to assess framework outcomes. [file 40814_2022_1017_MOESM4_ESM.docx]

## SUPPLEMENTARY FILE 4

Application of mixed method data to assess framework outcomes

| **Implementation Outcomes** | **Outcome Description** | **Type of Collected Data** | **Description of the Data Used to Evaluate the Outcome** | **Outcome Evaluation*** |
| --- | --- | --- | --- | --- |
| Feasibility | Extent to which various intervention components and procedures were successfully delivered in the setting | Quantitative data on number of disruptions | Number of times the intervention is interrupted | Number of disruptions < 2 for > 75% of children |
|  |  | Quantitative data on the cost of intervention (time or money) | Available time for the hypnotherapy screening and intervention | Sufficient time to deliver the intervention in > 80% of children who commence the hypnotherapy |
|  |  | - Consistency of implementation across staff/time (about process, not differential outcomes) - Qualitative data to understand implementation | Field notes on the adequacy of resources | Facilitators to the delivery to intervention in the context > barriers |
| Acceptability | Extent to which procedures were perceived as fit, satisfying and appealing, based on experienced affective and cognitive reactions | Quantitative data on the level of families’ satisfaction | Rating of families’ satisfaction with health evaluations and intervention on NRS | > 50% of families with moderate to high satisfaction rate (> 5 on 0 - 10 NRS) |
|  |  | Data on broader acceptability outcomes such as perceived benefits and burden, views and attitude towards the intervention. | Semi-structured interview with families on perceived required time and cognitive effort for the intervention | < 50% of children with high level of perceived burden |
|  |  |  | Semi-structured interview with parents, children and clinicians on likes, dislikes, recommendations. | Number of times themes related to acceptability emerge > number of times themes related to lack of acceptability across the interviews |
|  |  | Quantitative data on the level of children’s self-efficacy | Ratings of children’s perceived self-efficacy on Likert scale | > 50% of children with moderate to high score (5 to 10 on self-efficacy NRS) |
|  |  | Quantitative data on the level of children’s therapy expectations | Ratings of children’s positive therapy expectation level on Likert scale | > 50% of children with moderate to high score (5 to 10 on therapy expectations NRS) |
| Reach | Percent of potentially eligible participants who: a) declined, b) took part and c) have a positive attitude towards participation? | Quantitative data on the percentage of families willing to participate | Rate of eligible families who consented to participate and who received the intervention | > 50% of approached families consenting to participate and > 80% of participants who consent to the study receive the intervention. |
| Effectiveness | The impact of the intervention and study procedures on a) all participants who joined the program; b) on health outcomes; and c) on both positive and negative (unintended) outcomes. | Qualitative data on broader outcomes such as benefits and adverse effects. | Reports of parents, children and clinicians on adverse events. | - No serious adverse events and few (n ≤ 2) non-serious adverse events** - Number of perceived benefits > number of reported adverse events. |
|  |  |  | Perceived benefits of the intervention by children, caregivers and health professionals on perceived benefits | Perceived positive effects of the intervention > negative effects of the intervention based on interview data |
|  |  | Quantitative data on primary health outcomes | Pain, anxiety and itch ratings | > 50% of children with low to moderate procedural and post-procedural pain (< 5 on FPS-R/ NRSI and NRSU and FLACC), state anxiety (0 -50 mm on VAS) and itch scores (< 5 on itch-NRS) |
| Adoption | Percent of eligible clinicians in the setting (i.e., those conducting dressing changes of child participants) who participated. | Quantitative data on clinicians willing to participate | Report on the rate of eligible clinicians who consented to participate | > 50% of eligible clinicians consented to participate |
| Implementation | Extent to which the various intervention components were delivered as intended and as described in the protocol (i.e., fidelity) | Quantitative data on percentage of consistent delivery as required and completed data collection (e.g., adherence or consistency) | Report on attrition rate: number of child participants completing the intervention | > 50% of eligible children completed the intervention. |
|  |  |  | Adherence to the fidelity checklist in delivering the intervention | - Hypnotherapist adhered to > 50% of elements in fidelity checklist with > 50% of children. |
|  |  |  | Completeness of collected health outcomes data | - Health outcomes data collected with > 95% of participants who undergo hypnotherapy |
| NRS: Numeric Rating Scale; FPS-R: Faces Pain Scale-Revised; NRSI: Numerical Rating Scale for Pain Intensity; NRSU: Numerical Rating Scale for Pain Unpleasantness; VAS: Visual Analog Scale.  *Outcome evaluation: positive/negative outcome assessment using the available data.  **A serious adverse event, is an event that can be fatal, life-threatening, necessitating hospitalisation or extended hospital-stay, causing permanent or major disability, altering considerably life-functions, or leading to genetic abnormality or birth defect. A medical adverse event, that is not fatal nor life-threatening, nor requiring hospitalisation can be considered non-serious if they endanger the person or necessitate medical or surgical intervention to prevent the consequences of serious adverse events [1]. N.B. Maintenance will not be assessed due to absence of follow-up | | | | |

1. U.S. National Library of Medicine, U.S. National Institutes of Health, U.S. Department of Human Services. About the results database. 2016 [Available from: <https://clinicaltrials.gov/ct2/about-site/results>]
